# Atorvastatin treatment attenuates renal injury in an experimental model of ischemia–reperfusion in rats

**DOI:** 10.1186/1471-2369-15-14

**Published:** 2014-01-15

**Authors:** Kefei Wu, Wenjing Lei, Jianwei Tian, Hongyan Li

**Affiliations:** 1 Division of Nephrology, Huadu Hospital, Southern Medical University, Guangzhou, People’s Republic of China; 2 Institute of Nephrology Diseases, Nanfang Hospital, Southern Medical University, Guangzhou, People’s Republic of China

## Abstract

**Background:**

Recent studies in animal models have shown that statins can protect against renal failure independent of their lipid-lowering actions, and there is also an association between statin use and improved renal function after suprarenal aortic clamping. We investigated the hypothesis that post-ischemic acute renal failure could be ameliorated with atorvastatin (ATO) treatment and the possible molecular mechanisms in a model of ischemia–reperfusion (IR) in rats.

**Methods:**

Twenty-four male Sprague–Dawley rats were divided into three groups: sham, IR, and IR + ATO. ATO was given by a single intraperitoneal injection (10 mg/kg) 30 min before reperfusion in the IR + ATO group. The IR group and sham group received saline vehicle via the intraperitoneal route.

**Results:**

After 24 h of IR, serum creatinine levels were increased in the IR group compared with the sham group (p < 0.001). ATO treatment reduced the elevation of serum creatinine level by 18% (p < 0.05) and significantly increased the creatinine clearance rate (p < 0.001). Concentrations of advanced oxidation protein products and malondialdehyde were reduced in the ATO group, approaching levels observed in sham-group rats. ATO treatment alleviated pathological changes in renal tubular cells. Protein and mRNA levels of intercellular adhesion molecule-1 and monocyte chemotactic protein-1 were reduced significantly.

**Conclusions:**

These data suggest that direct protection of injured kidneys by ATO was possible even though the drug was injected 30 min before reperfusion, and that ATO may reduce IR injury by anti-inflammatory effects and by reducing oxidation stress.

## Background

The major causes of acute renal failure (ARF) are acute renal ischemia–reperfusion injury due to hemorrhage, severe injury, shock, or kidney transplantation. The mortality associated with ARF in critically ill patients remains high even though application of intensive care and blood purification has provided advanced treatment and a better prognosis for ARF patients [1,2]. Various agents, including dopamine, atrial natriuretic peptide, insulin-like growth factor, and endothelial receptor antagonists have been found to be effective in animals but ineffective in clinical studies [3-6]. It is necessary to explore and find more efficient and practical methods for timely prevention and cure.

ARF is a complex disorder resulting from heterogeneous pathogenic factors. Recent studies have shown that reactive oxygen species (ROS), inflammatory mediators such as intracellular adhesion molecule (ICAM-1) and monocyte chemotactic protein-1 (MCP-1) [7,8] as well as infiltration of inflammatory cells are implicated in renal ischemia–reperfusion injury [2]. In several studies, the effects of statins in ischemia–reperfusion models of renal protection have been related to their anti-inflammatory, antioxidant, and vascular actions [9-11]. Although these findings suggest an association between statin use and preserved renal function in ARF scenarios such as suprarenal aortic clamping, kidney transplantation, and critically ill patients in the intensive care unit [12,13], they carry little significance in patients with unpredictable ARF caused by hemorrhage, severe injury, or shock. In these studies, statin intervention has been several days before ischemia–reperfusion injury. However, the effects of statin intervention after ischemia-reperfusion injury are not fully understood and it is of significance to the clinical treatment of ARF. Todorovic et al. reported that a single intravenous bolus injection of simvastatin after ischemia could relieve tubular necrosis, suggesting that intervention after ARF maybe improve the prognosis [14]. Therefore, the present study was undertaken to examine the effects and mechanisms of atorvastatin (ATO) after renal ischemia–reperfusion injury with focuses on tubular damage, necrosis, inflammation and oxidant stress in rats.

## Methods

All animal procedures were approved by the Animal Experiment Committee of Southern Medical University (Guangzhou City, China).

### Animals

Male Sprague–Dawley rats (initial weight, 200–250 g; Southern Medical University Animal Experiment Center, Guangzhou City, China) were maintained under standardized conditions and fed a standard rodent diet, with free access to tap water.

### Model of ischemia–reperfusion injury

Surgery was undertaken with general anesthesia with 3% napental (30 mg/kg body weight (BW); Southern Medical University Reagent Center). Surgery was performed as described previously [15]. Bilateral flank incisions were made and the left renal pedicle occluded for 60 min, during which time a right nephrectomy was carried out.

The treatment group, i.e., the ischemia–reperfusion + atorvastatin (IR + ATO) group of rats, was given single-dose intraperitoneal ATO (10 mg/kg BW) 30 min before reperfusion. The control group (IR group) received saline vehicle via the intraperitoneal route and underwent a clipping procedure and contralateral nephrectomy as described above. The third group received saline treatment and underwent a sham operation (sham group). That is, bilateral flank incisions were made, the left renal pedicle dissected (but not clamped), and the right kidney removed. Afterwards, the incisions were closed and animals was allowed to recover.

### Samples

All animals were anesthetized for 60 min and killed 24 h later. Each group contained eight rats. Blood samples were obtained before surgery and at the time of killing. Urine samples were obtained from metabolism cages 24 h after surgery. Rats were perfused, *via* the aorta, with 100 mL of cold phosphate-buffered saline, and their kidneys removed.

### Measurement of serum levels of creatinine (Scr) and the creatinine clearance rate (Ccr)

Scr levels were measured and the Ccr calculated using a Toshiba Automatic Biochemistry Analyzer (Toshiba, Tokyo, Japan). Ccr was calculated on the basis of urinary creatinine, serum creatinine, urine volume, and body weight using the following formula [16]:

$$\text{Ccr = urinary creatinine}\left(\text{µmol/L}\right)\times \text{urinary volume (mL/kg/min)/Scr}\left(\text{µmol/L}\right).$$

### Morphological examination

Tissue for light microscopy was fixed in 10% phosphate-buffered formalin and embedded in paraffin. Five-micrometer-thick sections were processed for staining using periodic acid-Schiff and hematoxylin and eosin (H&E). Morphological analyses were undertaken by an experienced pathologist blinded to the tissue source. The following parameters were chosen to indicate morphological damage to the kidney after ischemia–reperfusion: loss of brush border; formation of tubular casts; tubular dilatation. These parameters were evaluated on a scale of 0–4: not present (0); mild (1); moderate (2); severe (3); very severe (4). Tubular necrosis was graded from none (0) to confluent (4). The extent of cortical necrosis was graded from 0 to 3: no necrosis (0); necrosis confined to the inner third of the cortex (primarily in the S3 segment of the proximal tubules) (1); necrosis extending into the upper third of the cortex (2); extensive necrosis of all areas of the cortex and in which necrotic tubules are present near the kidney surface (3). Each parameter was determined on six or more rats.

### Measurement of advanced oxidation protein products (AOPPs)

Plasma levels of AOPP were determined as described previously [17]. To exclude the interference of the turbidity of lipids upon light absorption, samples were diluted 1:5 in PBS and centrifuged (10,000 × g, 1 h, 4°C). Samples below the lipid layer were used for AOPP measurement.

### Measurement of malondialdehyde (MDA) levels

The MDA level in the serum was detected using a method described previously [18] and spectrophotometry (UV–Vis Spectrometer 6105; Jenway, Stone, UK). About 2.5 mL of trichloroacetic acid was added to 0.5 mL plasma and the tube left to stand for 10 min at room temperature. After centrifugation at 3500 rpm for 10 min, the supernatant was decanted and the precipitate washed with sulfuric acid. Then, 2.5 mL sulfuric acid and 3 mL thiobarbituric acid (TBA) in sodium sulfate were added to this precipitate. The coupling of lipid peroxide with TBA was carried out by heating in a boiling water-bath for 30 min. After cooling in cold water, the resulting chromogen was extracted with 4 mL of n*-*butyl alcohol by vigorous shaking. Separation of the organic phase was facilitated by centrifugation at 3000 rpm for 10 min and its absorbance at 530 nm determined.

### Immunohistochemical analyses

Expression of ICAM-1 and MCP-1 was assessed by immunohistochemical analyses as described previously [19]. Sections (thickness, 4 mm) were cut from paraffin blocks, mounted on polylysine-coated slides, and stained (H&E) for light microscopy. Sections were also dewaxed in xylene, rehydrated in a descending series of alcohols, and blocked for endogenous peroxidase and avidin/biotin activities. After blocking with 3% bovine serum albumin (BSA) in phosphate-buffered saline (PBS), sections were incubated with a rabbit polyclonal anti-mouse antibody against ICAM-1 and MCP-1 (1:150 dilution; Santa Cruz Biotechnology, Santa Cruz, CA, USA) overnight at 4°C. Sections were then washed and incubated with a secondary antibody (goat anti-rabbit) using the Evision + Labeled Polymer kit (Dako, Glostrup, Denmark) for 30 min followed by incubation with avidin-biotin–peroxidase complex (Dako) and development with diaminobenzidine chromogen for 5 min. Finally, sections were rinsed in distilled water, counterstained with hematoxylin (Dako), and mounted on glass slides before evaluation under the microscope. Semi-quantitative analyses of immunohistochemistry were undertaken by image analysis software (Image Pro Plus; Media Cybernetics, Rockville, MD, USA).

### Reverse transcriptase-polymerase chain reaction (RT-PCR)

Detection of ICAM-1 and MCP-1 mRNA in renal tissue was undertaken. Briefly, total RNA of the renal tissue samples was extracted with an RNA Extraction Agent (TaKaRa Bio, Shiga, Japan) following manufacturer instructions. cDNA was synthesized using a TaKaRa Bio Reverse Transcription kit following manufacturer instructions. RT-PCR was performed using the SYBR Green kit of the TaKaRa Bio Reaction System according to the manufacturer’s instructions. The primers for ICAM-1 were: sense, 5′-GCGACCACGGAGCCAATTTCTCAT-3′; anti-sense, 5′-TCAGGACCCTAGTCGGAAGATCGAA-3′. The primers for MCP-1 were: sense, 5′-ACGCTTCTGGGCCTGTTGTTCA-3′; anti-sense,5′-TGGGGCATTAACTGCATCTGGCT-3′; glyceraldehyde 3-phosphate dehydrogenase was used as the internal control. The result (Ct) from ABI7500 Fast system was analyzed and compared using the 2^-△△Ct^ method.

### Statistical analyses

One-way analysis of variance (ANOVA) was used to compare the different mean values in the three experimental groups. The least-square difference or Dunnett’s T3 test (equal variance not assumed) was used to ascertain significant differences among the groups. Scr was analyzed by repeated-measures ANOVA followed by Dunnett’s T3 multiple comparison test. Histological evaluation was assessed by the Kruskall–Wallis test followed by the Rank sum test for multiple comparison. p < 0.05 was considered significant. Data are the mean ± SD. SPSS version 13.0 (SPSS, Chicago, IL, USA) was used for all analyses.

## Results

### Scr levels were decreased and Ccr levels increased in the IR + ATO group

**Figure 1:**
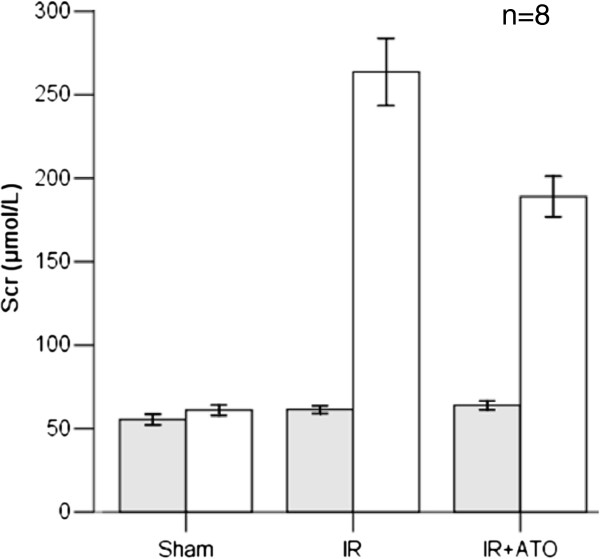
**Scr before (shaded square symbol) and 24 h after (□) ischemia–reperfusion injury.** Ischemia–reperfusion injury and treatment with saline vehicle resulted in significant elevation of the Scr level after 24 h. Treatment with ATO after ischemia–reperfusion injury (IR + ATO) reduced the impairment of kidney function compared with the IR group (one-way ANOVA test; p < 0.05; n = 8). A right nephrectomy without ischemia of the contralateral kidney (sham) did not increase the Scr level after 24 h.

Sixty minutes of ischemia and subsequent reperfusion caused a decline in renal function, and Scr levels were increased significantly, compared with the sham-operated group (270.75 ± 46.84 μmol/L vs 61.23 ± 8.49 μmol/L; p < 0.001) (Figure 1). Decreases in renal function were ameliorated by ATO treatment, which demonstrated increases in Scr levels 24 h after surgery (189.00 ± 31.85 μmol/L), compared with sham-operated animals (p < 0.05). Right nephrectomy alone (sham operation) did not increase creatinine levels.

**Figure 2:**
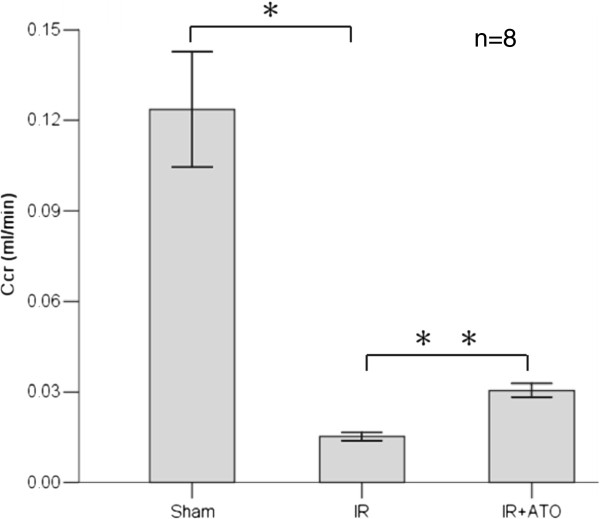
**Ccr after 60 min of ischemia without treatment (IR) or with ATO pretreatment (IR + ATO), compared with sham-operated control animals (sham).** IR induced decreases in the Ccr (one-way ANOVA test; *p < 0.001, compared with the sham group; n = 8). Treatment with ATO increased the Ccr (one-way ANOVA test; **p < 0.001, compared with the IR group; n = 8).

To substantiate the effects of ATO treatment on renal function after ischemia, we analyzed the Ccr (Figure 2). The Ccr in sham-operated animals was 0.127 ± 0.034 mL/min and reduced to 0.015 ± 0.004 mL/min after ischemia–reperfusion injury. ATO significantly ameliorated these decreases (0.031 ± 0.006 mL/min; p < 0.001).

### Pathological changes in renal tubular cells were alleviated in IR + ATO groups

**Figure 3:**
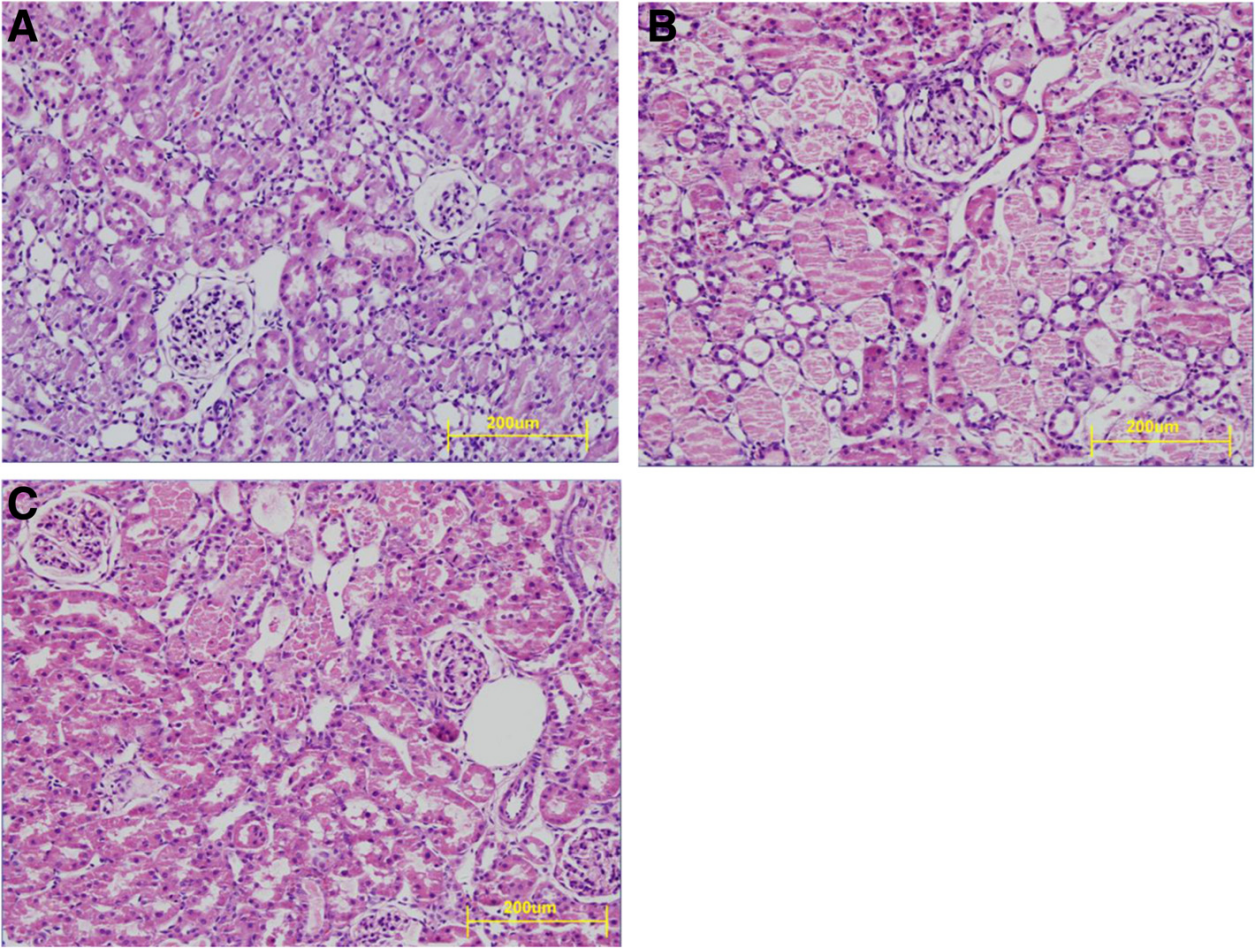
**Morphological damage in the renal cortex of proximal tubules.** Ischemia–reperfusion caused severe damage, with loss of the brush border, tubular dilatation, detachment of epithelial cells from the basement membrane, and necrosis of tubular casts. **(B)** ATO treatment (IR + ATO) markedly reduced the pathological changes noted above. **(C)** Control animals after right nephrectomy without ischemia (sham) exhibited almost no abnormalities in the contralateral kidney. **(A)** H&E. Magnification, ×40 in A–C.

The morphological changes in tissues during different experiments are shown in Table 1. Representative examples of these experiments are presented in Figure 3. Ischemia–reperfusion injury caused severe damage to epithelial cells of the proximal tubule in the renal cortex. Loss of the brush border and detachment of epithelial cells from the basement membrane caused tubular obstruction, tubular casts, and necrosis (Figure 3B). After ATO treatment, tubular necrosis was markedly reduced and the pathological changes described above mitigated (Figure 3C). Kidneys from sham-operated rats are shown in Figure 3A.

**Table 1:** Quantitative evaluation of morphological kidney damage

**Group**	**N**	**Brush border loss**	**Tubule dilatation**	**Casts**	**Necrosis**	**Extent of necrosis**
Sham	6	3.75^a^	3.92^a^	3.83^a^	3.67^a^	4.25^a^
IR	6	15.33^a^	15.33^a^	15.00^a^	15.42^a^	14.50^a^
IR + ATO	6	9.42^a^	9.25^a^	9.67^a^	9.42^a^	9.75^a^

Histologic evaluation of kidney after renal ischemia.

Morphology was scored according to loss of brush borders of proximal tubules, cast formation within tubules, tubule dilatation, and tubule necrosis. Kruskall–Wallis test. Values represent mean rank. ^a^p < 0.05; n = 6. There is a significant difference between the three groups and according to the rank sum test, and multiple comparison test (values represent the mean, subset for alpha = 0.05).

### Expression of AOPP and MDA were down-regulated in IR + ATO groups

**Figure 4:**
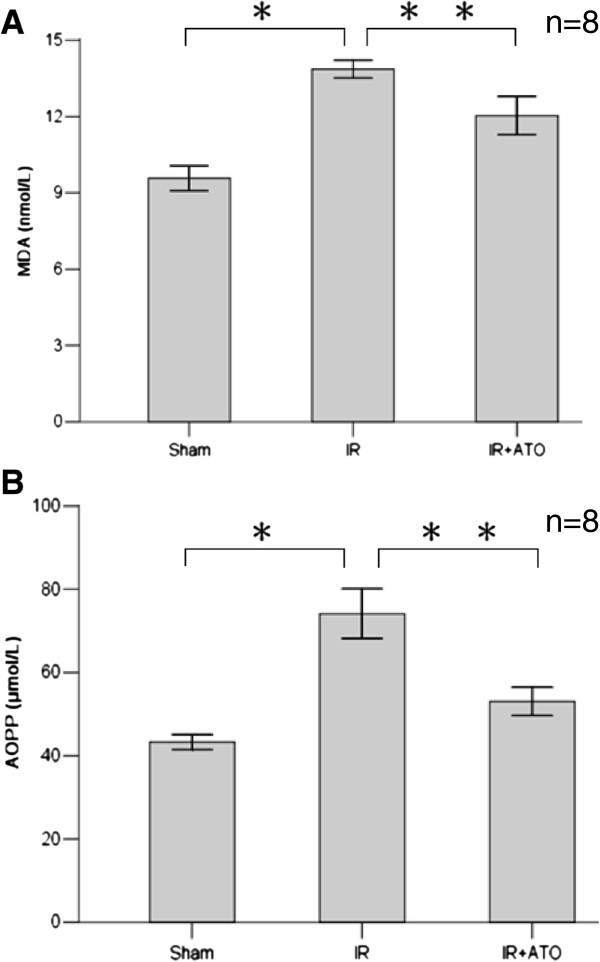
**AOPP and MDA levels in different groups. A** Ischemia–reperfusion induced increases in AOPP levels (one-way ANOVA test; *p < 0.001, compared with the sham group; n = 8). Treatment with ATO significantly decreased AOPP levels (**p < 0.005; compared with the IR group; n = 8). **B** MDA levels after ischemia–reperfusion were increased (one-way ANOVA test; *p < 0.001, compared with the sham group; n = 8) and were decreased after administration of ATO (**p < 0.05, compared with the ischemia–reperfusion group; n = 8).

AOPP concentrations in the IR group (74.13 ± 16.81 μmol/L) differed significantly (p < 0.001) from those in the sham group (43.25 ± 5.10 μmol/L) and the ATO group (53.03 ± 8.99 μmol/L). AOPP levels were significantly decreased in the ATO group compared with the IR group (p < 0.005) (Figure 4A). Serum levels of MDA in the IR group (13.86 ± 0.99 nmol/L) differed significantly from those in the sham group (9.57 ± 1.41 nmol/L) and the ATO group (12.04 ± 1.97 nmol/L). They were decreased significantly in the ATO group compared with the IR group (p < 0.05) (Figure 4B).

### Protein and mRNA levels of ICAM-1 and MCP-1 were reduced in IR + ATO groups

**Figure 5:**
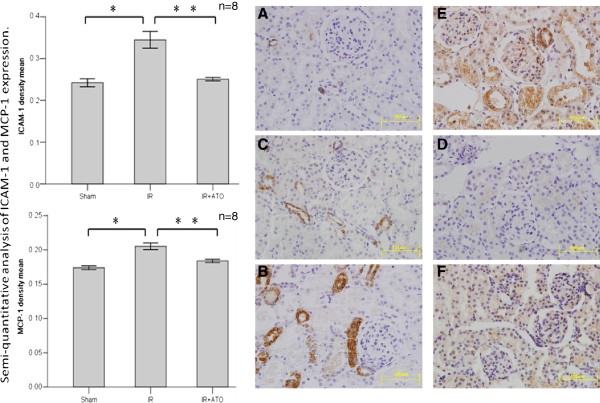
**Representative immunohistochemical staining profiles of expression of ICAM-1 and MCP-1 at 24 h and the results of semi-quantitative analyses.** Ischemia–reperfusion **(A and D)** caused significant expression of ICAM-1 and MCP-1 in renal tubules and the cortical peritubular interstitium **(B and E)**. Atorvastatin (IR + ATO) reduced this expression **(C and F)**. Magnification, **×**400 in A–F. Expression of ICAM-1 and MCP-1 after ischemia–reperfusion was increased (one-way ANOVA test; *p < 0.05, compared with the sham group; n = 8) and decreased after administration of ATO (**p < 0.05, compared with the IR group; n = 8).

We addressed the effects of ATO on expression of ICAM-1 and MCP-1. Post-ischemic changes were reflected by the upregulation of adhesion molecules. Representative immunohistologic-stained specimens are shown in Figure 5.

Ischemia–reperfusion injury induced massive upregulation of expression of ICAM-1 and MCP-1 in renal tubules and the cortical peritubular interstitium (Figures 5B and E). ATO blocked ICAM-1 upregulation in the renal tubule and cortical peritubular interstitium (Figure 5C), as well as perivascular and endothelial upregulation of MCP-1 expression (Figure 5F). Expression of ICAM-1 and MCP-1 in control sections from the corresponding areas in the kidneys of sham-operated rats is shown in Figures 5A and D. Ischemia–reperfusion injury caused significant elevation of expression of ICAM-1 and MCP-1, and ATO decreased the upregulation of the expression of ICAM-1 and MCP-1 in the IR group (p < 0.05) (Figure 5).

**Figure 6:**
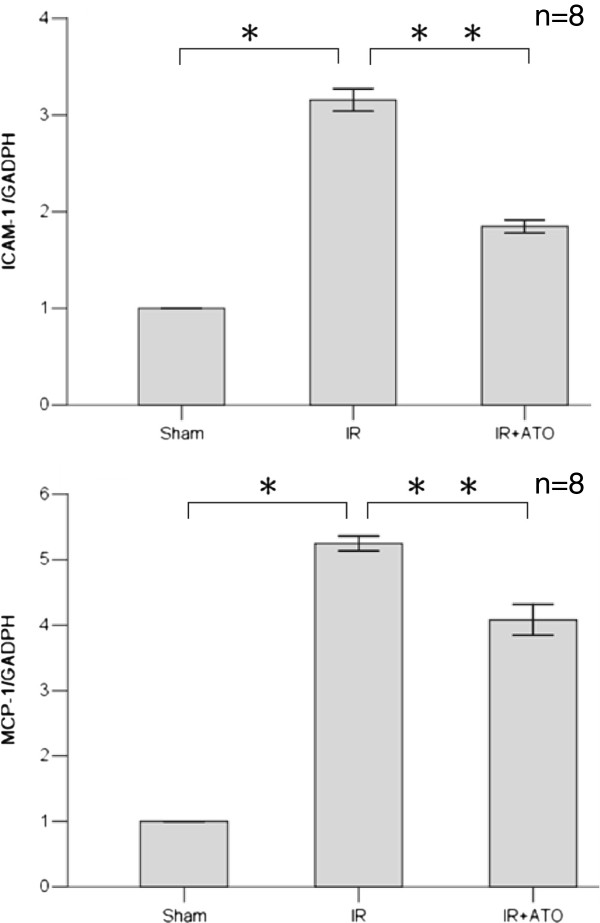
**Quantitative RT-PCR analyses of ICAM-1 mRNA and MCP-1 mRNA gene expression in kidneys.** Ischemia–reperfusion caused severe increase in expression of ICAM-1 and MCP-1 mRNAs (one-way ANOVA test; *p < 0.001 compared with the sham group; n = 8). ATO reduced this upregulation (**p < 0.001, compared with the IR group; n = 8).

Next, we examined the mRNA changes of ICAM-1 and MCP-1 in the kidneys of rats from each group. Ischemia–reperfusion injury caused significant elevation of expression of ICAM-1 and MCP-1 (Figure 6). ATO decreased the upregulation of expression of ICAM-1 and MCP-1 mRNA in the IR group. The kidneys of the sham-operation group showed extremely low expression of these molecules.

## Discussion

The present study demonstrated that, according to the selected biochemical and histological parameters of renal dysfunction, ATO treatment 30 min after ischemia mitigated the course of ischemic ARF in rats. This observation was supported by several key findings. ATO treatment 30 min after ischemia significantly ameliorated post-ischemic acute tubular necrosis and considerably limited the structural damage after ischemia. Histologically, ATO treatment reduced the damage to proximal tubules in the renal cortex. Untreated animals demonstrated typical changes: loss of the brush border, destruction of epithelial cells, “naked” basement membranes, and tubular obstruction. The major changes in tubules (loss of nuclei and appearance of tubular debris and casts) were remarkable. Moreover, ATO treatment reduced the serum levels of creatinine and increased the Ccr, which are indicators of impaired glomerular function. As mentioned above, despite intervention being after ischemic injury, ARF could be ameliorated with statin treatment.

There are several possible mechanisms for the protective effects of ATO. Recent studies have shown that ROS, certain inflammatory mediators (e.g., ICAM-1, MCP-1 [7,8]), and infiltration of inflammatory cells are related to renal ischemia–reperfusion injury [9]. During ischemia–reperfusion injury, reperfused tissues generate a great deal of ROS through activation of xanthine oxidase and NADPH oxidase. ROS not only elicit injury to reperfused tissue directly, they also amplify the effect of injury of ROS through peroxidation (oxidative stress). This view is consistent with the notion that oxidative stress increases in critically ill patients with acute kidney injury (AKI) [20].

AOPPs are derived from oxidation-modified albumin (its aggregates or fragments), and are recognized as markers of oxidative damage to proteins, the intensity of oxidative stress, and inflammation [21]. Accumulation of AOPPs is probably *via* a redox-sensitive inflammatory pathway that causes deterioration in renal dysfunction [22] and it is also a prognostic biomarker for recovery from AKI after coronary artery bypass grafting [23]. Furthermore, ROS also cause renal-cell injury by lipid peroxidation, which results in increased membrane permeability in cells, mitochondria, and lysosomes. MDA is one of several low-molecular-weight end products formed *via* the decomposition of certain primary and secondary lipid peroxidation products [24]. MDA is a reliable estimator of lipid peroxidation [25].

We found that ischemia–reperfusion injury led to an increase in levels of AOPP and MDA that were decreased by ATO treatment. These results suggest that the protection afforded by ATO may be mediated by reducing oxidative stress, particularly by decreasing the production of oxygen free radicals and lipid peroxidation in cells.

Inflammation is another important factor causing ischemia–reperfusion renal damage. Infiltration of inflammatory cells in ischemia–reperfusion injury is accompanied by significant upregulation of expression of adhesion molecules [7,8]. Upregulation of expression of ICAM-1 and MCP-1 in glomeruli, renal tubules, and the peritubular interstitium was also prevented by ATO treatment. Our results showed that even administration of ATO after ischemic ARF elicited anti-inflammatory actions.

The most prominent injury to renal proximal tubular cells is likely to occur during the first 2–4 h of the reperfusion period. Sixty minutes of ischemia does not increase MDA levels, whereas 15 min of reperfusion results in a large increase in kidney lipid peroxidation and aggravates cell injury [14,15]. Hence, ATO administration 30 min before reperfusion can protect the kidney.

In other studies, statin intervention has been several days before ischemia–reperfusion injury [26,27] but, in the present study, drug intervention was after renal ischemia. AKI is an unpredictable event, so the present study has more clinical relevance compared with previous studies. Haylor et al. reported that even if ATO was administered after clamping the renal hilus but before kidney reperfusion, renal ischemia–reperfusion injury can be improved because of direct inhibition of activated caspase-3 in rats [28]. Our results are consistent with the findings of that study but we studied whether ATO treatment after renal ischemia also had anti-inflammatory and anti-oxidative stress effects. In their study, ATO did not change Scr levels but in the present study Scr levels were decreased and Ccr levels increased in ATO groups. This finding may be because we determined Scr levels 24 h after reperfusion, whereas in their study it was at 4 h. Scr levels and the Ccr are the most important indices of renal function. Taken together, the renal protection afforded by ATO is not obvious 4 h after reperfusion but its effects continue for ≥4 h. The present study showed that renal function could obviously recover in 24 h even if ATO was given after ischemia.

## Conclusions

We demonstrated that ATO given after renal ischemia has anti-inflammatory and anti-oxidative stress effects. Treatment of ATO ameliorated renal ischemia–reperfusion injury in vivo. Levels of Scr, AOPP, MDA, ICAM-1, and MCP-1 were reduced but the Ccr increased. Our results suggest that acute administration of ATO after ARF can reduce renal damage and promote recovery of kidney function.

## Competing interests

The authors declare that they have no competing interests.

## Authors’ contributions

HY conceived of the study, participated in its design and coordination, and helped to draft the manuscript. KF participated in the design of the study, carried out the laboratory experiments, analyzed the data, interpreted the results, and wrote the manuscript. JW and WJ carried out the laboratory experiments. All authors approved the final version of the manuscript.

## Pre-publication history

The pre-publication history for this paper can be accessed here:

http://www.biomedcentral.com/1471-2369/15/14/prepub
